# Evaluating equity interventions and the limits of institutional change: from admission to clinical leadership in UK medical education

**DOI:** 10.3389/fmed.2026.1895111

**Published:** 2026-08-12

**Authors:** Carl Kulimushi, Rachel Cowen

**Affiliations:** Division of Medical Education, School of Medical Sciences, Faculty of Biology, Medicine and Health, University of Manchester, Manchester, United Kingdom

**Keywords:** belonging, contextual admissions, equity, gender equity, medical education, reverse mentoring, structural inequality, widening participation

## Abstract

Structural inequality in UK medical education does not arise at a single point. It builds across the career pipeline, from application through to clinical leadership, and current institutional responses address it only in part. This Mini Review examines one widely implemented equity intervention at each of three pipeline stages: contextual admissions at access, reverse mentoring at success, and equity-focused leadership and fellowship schemes at progression. The review applies the Equity Evaluation Framework, which assesses interventions against three criteria: theoretical grounding, structural and cultural impact, and equity and belonging. The same finding recurs at each stage. Interventions change practice at a defined point in the pipeline, while the governance arrangements, accountability systems, and professional cultures that produce inequality remain largely unaltered. The available evidence is constrained by cross-sectional designs, limited intersectional analysis, and a focus on participation rates rather than structural outcomes. The review argues that incremental interventions at the margins of the pipeline are unlikely to deliver equity on their own, and that durable change depends on embedding equity within institutional governance and culture at every stage.

## Introduction

Structural inequality in UK medical education is not a problem of access alone. It builds across the whole career pipeline, from application through to clinical leadership, and existing institutional responses have addressed it only in part. Despite formal commitments to widening participation, diversity, and inclusion, students and professionals from socioeconomically disadvantaged, racially minoritised, and underrepresented gender groups continue to meet barriers that compound at successive career stages (1–4). The consequences reach beyond individual careers, because workforce composition affects patient outcomes, institutional culture, and wider health equity (5–7).

The questions of who enters medicine, who succeeds within it, and who advances into leadership are connected, because the rules governing admissions, the cultures shaping student experience, and the norms determining who reaches senior roles draw on the same underlying inequalities. Examining why equity interventions often fall short therefore requires attention to what they do and to what they leave unchanged.

This Mini Review examines equity-focused interventions across three stages of the UK medical education and clinical career pipeline: access, success, and progression. Using a narrative, comparative case study approach, it evaluates one widely implemented intervention at each stage and asks whether interventions increase representation and whether they produce the structural and cultural change that makes representation durable.

## Background

**Figure 1:**
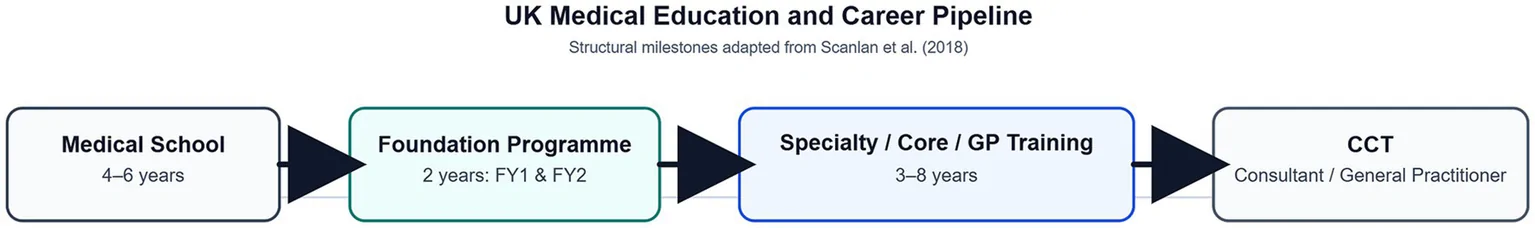
Medical education and career pipeline in the United Kingdom. The figure illustrates the journey from medical school application to clinical practice and leadership, and identifies transition points where opportunities and outcomes may diverge (10).

The medical education and career pipeline in the United Kingdom runs from school-age aspiration to clinical leadership, and inequality operates at every stage. Disadvantage accumulates rather than appearing in isolation, and it shapes who enters medical school, who succeeds, and who advances into senior roles. Applicants from widening participation backgrounds meet barriers rooted in unequal social, economic, and cultural capital, and that disadvantage persists after admission through financial strain and exclusion from informal networks (8, 9). Racially minoritised students meet systemic barriers including exclusionary curricula and racism, with measurable consequences for attainment (1, 2). Gender disparities extend further. Despite near-parity at student level, women hold only 20% of Editor-in-Chief positions at leading medical journals (3, 4). Figure 1 maps this journey from application to leadership and identifies the transition points where divergence occurs (10).

Four related concepts frame the analysis. Equity concerns the structural removal of barriers through institutional change, while diversity describes demographic presence, which does not by itself produce meaningful inclusion (11, 12). Inclusion requires that marginalised groups are actively valued rather than merely represented, and belonging describes the psychological safety that makes full participation possible (12, 13). Intersectionality applies across all four concepts, because overlapping identities compound disadvantage at every pipeline stage and move the focus from representation toward power (14). Following Rehman et al., intersectionality is understood here not as an additive model in which categories of disadvantage simply accumulate, but as a transformative one in which overlapping identities reshape the character of disadvantage at each stage. The three case studies that follow are separated analytically for clarity; they do not imply that socioeconomic, racial, and gender inequalities operate independently across the pipeline.

To evaluate whether interventions address these dynamics or only manage their appearance, this review applies the Equity Evaluation Framework. The Framework was developed for this review by drawing together principles from equity scholarship and constructivist learning theory (15, 16). Constructivism holds that educational outcomes are socially mediated rather than individually determined, so interventions need to target the conditions that produce inequality rather than its surface effects. The Framework applies three criteria, summarised in Table 1: theoretical grounding, which asks whether an intervention targets structural conditions rather than individual deficit; structural and cultural impact, which assesses whether it reaches formal processes including governance, assessment, and promotion; and equity and belonging, which evaluates whether it supports sustained participation and clear institutional accountability.

**Table 1:** Equity evaluation framework: criteria for evaluating interventions across the access, success, and progression stages of the medical education and career progression pipeline.

Criterion	What the criterion assesses
Theoretical grounding	Whether the intervention targets the structural conditions that produce inequality rather than a presumed deficit in the individual.
Structural and cultural impact	Whether the intervention reaches formal institutional processes, including governance, assessment, and promotion, rather than operating only at the margins.
Equity and belonging	Whether the intervention supports sustained, valued participation and is underpinned by clear institutional accountability.

The table summarises the three criteria used to appraise interventions, covering theoretical foundations, structural impact, and capacity to support equity and belonging across the educational and career pathway.

Each stage of the review examines one illustrative case study: contextual admissions at access, where socioeconomic status remains the most entrenched barrier to entry (17, 18); reverse mentoring at success, with a focus on Black heritage students because aggregated ethnic categories obscure the distinct attainment gaps this group faces (19–21); and equity-focused leadership and fellowship schemes at progression, where despite numerical parity at entry women meet a well-documented pattern of attrition toward senior roles (22, 23). These cases do not exhaust the inequalities present across the pipeline. The experiences of disabled students and LGBT+ practitioners fall beyond the scope of this review, though it should be noted that the limited published evidence available for these groups is not a neutral gap; it reflects a wider failure of institutional data collection and research investment that compounds the very exclusions this review describes. Contextual admissions were selected at the access stage because socioeconomic status remains the most entrenched barrier to entry and the intervention is widely implemented across UK medical schools (18, 24). Reverse mentoring was selected at the success stage because, unlike deficit-focused interventions directed at the individual learner, it repositions student experience as a source of institutional learning, offering a stronger test of whether institutions translate critical dialogue into structural change (25, 26). Leadership and fellowship schemes were selected at the progression stage because gender represents the pipeline’s sharpest paradox: numerical parity at entry coexists with marked underrepresentation at senior levels, making it the clearest available case for examining whether representation alone produces durable structural change (22, 23). Each case was also chosen because it offers the strongest available evidence base for evaluation at that pipeline stage.

## Discussion

### Access: socioeconomic barriers and contextual admissions

Access to medical education in the United Kingdom remains shaped by socioeconomic inequality despite decades of widening participation policy. National data show that only 6.4% of UK medical students come from the most deprived areas, compared with 28% from the most advantaged quintile, and offer-rate gaps have changed little since 2012 (18). The admissions process itself reproduces this advantage. The UCAT, personal statements, and structured interviews reward prior access to coaching, cultural capital, and institutional guidance that lower socioeconomic status applicants rarely possess (24, 27). In 2021, applicants from the most deprived areas had odds of 0.55 of receiving an offer relative to the most advantaged peers, a gap that academic potential alone cannot explain (18). Coyle et al. argue that admissions systems construct merit discursively, reframing middle-class advantage as excellence and presenting structural privilege as neutrality (27).

Contextual admissions adjust entry thresholds using indicators such as postcode, school type, and care experience, in recognition that equal grades do not reflect equal potential (17, 28). The principle marks a shift from equality of treatment toward equity of outcome, but implementation remains uneven. A 2020 audit found wide variation in the indicators institutions used and in how flagged applicants were assessed, with limited transparency in decision-making (24). A HEPI report found that 65% of students were unsure whether contextual factors had influenced their offer, which weakens the legitimacy that belonging requires (29). Where post-admission support is absent, as Cleland et al. and Ravulapalli et al. both confirm, the intervention does not alter the conditions students meet once inside (30, 31). Against the Framework, contextual admissions show partial theoretical grounding, modest structural impact concentrated at entry, and weak alignment with equity and belonging after admission. The evidence also remains largely single-axis: applicants who are both socioeconomically disadvantaged and racially minoritised face compounding barriers that current contextual admissions data do not capture, because most schemes flag socioeconomic indicators alone (14, 24). Curtis et al. identify the central limitation, namely that many schemes target perceived student shortcomings rather than the systemic conditions that produce them (28). Widening the door is a different task from changing what lies beyond it.

### Success: racialised barriers and reverse mentoring

For Black heritage students, the conditions needed to succeed within medical education are not equally available. Adjusted UK Foundation Programme Z-score data confirm statistically significant attainment gaps relative to White peers, after controlling for background variables, and aggregated ethnic classifications have historically obscured these gaps (21). Gill and Mohamud both argue that broad categories mask distinct patterns of anti-Black racism and produce equity responses too diffuse to address the most urgent inequities (19, 20). Disaggregating the evidence is an ethical requirement rather than a methodological preference.

The conditions that produce this gap are institutional. The formal curriculum remains predominantly Eurocentric, and the hidden curriculum embeds inequity through professional norms within which Black students are disproportionately scrutinised (1, 32, 33). Racism and microaggressions are widespread and weaken psychological safety and professional identity development (2). Structural bias reaches into formal assessment. Brown et al. found that candidate race significantly influenced simulated patient ratings at fail and borderline thresholds, which shows discrimination operating within the mechanisms that formally determine progression (34). The cumulative effect produces identity strain and emotional exhaustion among students navigating these environments (35).

Reverse mentoring repositions student experience as institutional learning rather than individual deficit, and evidence from the Southampton scheme showed measurable shifts in senior staff perceptions (25). Israni’s systematic review confirms its potential when it is implemented with authentic intent and clear structural aims (36). Against the Framework, its theoretical grounding is strong. Its structural reach is more limited. Williams cautions that without embedding in curriculum reform and formal accountability, reverse mentoring functions as critical conversation without institutional consequence (37). Campbell and colleagues’ analysis of the minority tax is equally relevant, because without adequate support reverse mentoring places additional emotional labour on the students it is designed to benefit (38, 51). The intersectional dimension is again largely absent from evaluation: Black women and those from low-income backgrounds may bear a disproportionate share of this labour, yet the published evidence does not disaggregate outcomes along these lines (14). The intervention opens a channel for institutional learning, but whether institutions act on what they hear is a question it cannot answer alone.

### Progression: gendered exclusion and leadership schemes

Although Figure 1 maps the pipeline to CCT-level clinical practice, clinical academic leadership represents a downstream extension of that trajectory and remains subject to the same institutional forces; it is therefore included here as a further stage of the pipeline rather than a separate domain (22, 23). Women now comprise a majority of UK medical students, yet this parity does not hold at subsequent career transitions. Men still constitute 64.5% of the clinical academic workforce and 74.4% of clinical professors, and women hold only 20% of Editor-in-Chief positions at leading medical journals (3, 39). The same seniority gradient recurs across other demographics. In the NHS medical workforce in England, racially minoritised doctors comprise 63.2% of non-consultant grades but 42.1% of consultants, and hold 17.3% of trust board positions against 30.7% of the overall workforce (40), while ethnic disparities also persist at senior levels of clinical academia (39). Gendered attrition is examined here as the clearest documented instance of this wider pattern, consistent with the selection rationale set out above; progression data disaggregated by ethnicity or socioeconomic background, and above all by their intersection, remain markedly scarcer than for gender (14, 39). Brown et al. describe a leaky pipeline of disproportionate attrition at each transition that numerical diversity at entry does not prevent (23).

The mechanisms are both structural and cultural, and they reinforce one another. Inflexible training pathways disadvantage those with caregiving responsibilities, under-provision of mentorship compounds attrition at critical transitions, and informal networks shape promotions in ways that reproduce male-dominated leadership (22, 41). Cultural norms frame authority and research excellence through a male-normative lens that penalises non-linear careers, and Henderson and Bhopal argue that organisational climates rewarding constant visibility select against the career patterns that caregiving produces (41).

Athena Swan and NIHR Fellowship schemes have historically linked equity planning to research funding eligibility and expanded leadership development pathways. Applicants are now instead required to demonstrate a broader, generalised commitment to equality, diversity, and inclusion (EDI), with Athena Swan accreditation serving as one optional route. Ovseiko et al. found modest but statistically significant increases in women’s leadership representation in award-holding departments (42). Against the Framework, however, Caffrey et al. and Kalpazidou Schmidt et al. identify the same limitation, namely that institutional engagement frequently becomes compliance-driven, motivated by funding eligibility rather than genuine cultural reform (43, 44). Burkinshaw et al. note a further difficulty, in that the burden of sustaining these initiatives falls disproportionately on the women they are designed to support (45). Structural incentives can shift behaviour, but they have not yet shifted the culture that makes those incentives necessary. Comparable findings from mentorship research in other male-dominated professional fields point to the same limitation (52). That burden is unlikely to be evenly distributed: women from racially minoritised backgrounds and those with caregiving responsibilities face intersecting barriers that single-axis gender analyses do not capture (14, 46).

### Synthesis, gaps, and future directions

Across all three stages the same pattern holds. Interventions produce measurable change at specific points in the pipeline while the institutional conditions that generate inequality remain largely intact. This reflects a limitation of reach rather than of intent, and it is the pattern the Equity Evaluation Framework is designed to make visible, namely the difference between what an intervention does at the point of contact and what it leaves unchanged in governance, accountability, and professional culture. Contextual admissions, reverse mentoring, and fellowship schemes each represent genuine progress, but none, as currently implemented, amounts to structural transformation.

The implications for clinical practice are direct. Diverse clinical teams deliver safer, higher-quality care, particularly for diverse patient groups (5, 47). A socioeconomically representative workforce is associated with improved trust and health outcomes among underserved populations (38). Reverse mentoring supports cultural humility in senior clinicians, and systematic reviews link culturally competent education to reductions in racial disparities across clinical metrics (14). Institutions with higher gender diversity at leadership level show measurably improved hospital quality, including lower mortality (6). Workforce exclusion is therefore not confined to the training pipeline, because its consequences reach patients.

The evidence base has clear limitations. The selection of one intervention per pipeline stage also introduces potential selection bias; alternative interventions exist at each stage and may demonstrate different patterns of structural reach. Most evaluative studies are cross-sectional, single-site, and short-term, which makes it difficult to assess whether any intervention produces durable change across the pipeline (18, 25, 42). Longitudinal cohort data tracking outcomes from admission to leadership remain scarce, and intersectional analysis examining how socioeconomic, racial, and gender disadvantage compound across the pipeline is underdeveloped (14). The siloed structure of the available evidence base, in which outcomes are reported by single protected characteristic, is itself a structural limitation, constraining the conclusions that any review of this kind can draw. The review is UK-specific, which limits direct transferability, although the Framework is designed to apply to other regulated professional training contexts shaped by central oversight and competitive funding. Evaluation has also concentrated on representational outcomes, such as participation rates and attitudinal shifts, rather than on structural reach into governance, promotion criteria, or formal accountability.

A genuine controversy runs through the field. One position holds that incremental institutional reform, consistently implemented and properly resourced, can produce meaningful structural change over time. Another holds that the pipeline model reproduces the hierarchies it claims to address by treating entry, success, and progression as discrete technical problems rather than expressions of the same systemic inequality (27, 37, 43). Growing international pressure against diversity, equity, and inclusion programmes sharpens this tension, because where external accountability weakens, compliance fatigue can replace genuine reform, and UK medical education is not insulated from these pressures (48, 49). Abbasi describes progress in embedding racial equity in healthcare leadership as glacial (7), a pattern echoed in reporting on racial harassment complaints in UK medical schools, where rising complaint numbers may reflect either genuine progress or simply greater willingness to report (50).

Future research should prioritise longitudinal, intersectional evaluation that tracks outcomes across the full pipeline rather than at isolated points, and should assess interventions for their reach into formal governance and promotion criteria rather than for representational effects alone. Institutions need accountability mechanisms that reward cultural change rather than the production of action plans. Greater student and junior staff involvement in governance, beyond the consultative role that reverse mentoring currently provides, is an underexplored route toward the structural change the evidence repeatedly identifies as absent. The pipeline is unlikely to become equitable through better interventions at its margins. it will become equitable when the institutions that operate it treat equity as a condition of their legitimacy rather than as a programme to be managed, beginning with data and accountability systems capable of capturing intersectional rather than single-category outcomes.
